# Supplementary material for: 14-3-3 Proteins and the Plasma Membrane H+-ATPase Are Involved in Maize (Zea mays) Magnetic Induction
Source: Plants (Basel). 2023 Aug 7;12(15):2887. doi: 10.3390/plants12152887 (PMC10421175; doi:10.3390/plants12152887)
Supplement: Supplementary file 1 [file plants-12-02887-s001.zip › plants-2515579-supplementary.pdf]

Supplementary Table S1. Primers used in this work

Gene	Locus	Sequence 5' → 3'	
ACT1 - actin1	Zm00001d010159	ATGGATTCCGGTGATGGTGT	Forward
ACT1 - actin1	Zm00001d010159	ACGTAGGCAAGCTTCTCCTT	Reverse
ACT1 - actin1	Zm00001d010159	GCAGCATGAAGGTTAAAGTGATTG	Forward
ACT1 - actin1	Zm00001d010159	GCCACCGATCCAGACACTGT	Reverse
GAPC2 - glyceraldehyde-3-phosphate dehydrogenase2	Zm00001d035156	CACCGTGGATGTGTCAGTTG	Forward
GAPC2 - glyceraldehyde-3-phosphate dehydrogenase2	Zm00001d035156	AAACCAGATCCTCCTCCACG	Reverse
GAPC2 - glyceraldehyde-3-phosphate dehydrogenase2	Zm00001d035156	CATGCTGGTTTTGGACACGG	Forward
GAPC2 - glyceraldehyde-3-phosphate dehydrogenase2	Zm00001d035156	GCGAATTCGTTACAGGACGG	Reverse
α-tubulin - tubulin α-3 chain	Zm00001d013367	GCGCACCATCCAGTTCGT	Forward
α-tubulin - tubulin α-3 chain	Zm00001d013367	CTGGTAGTTGATTCCGCACTTG	Reverse
TUB1 - β tubulin1	Zm00001eb000490	TCGTGGGTGTGTTTCGTTTC	Forward
TUB1 - β tubulin1	Zm00001eb000490	CCAAAGCGCAACAGGGTATT	Reverse
ELFA2 - elongation factor alpha2	Zm00001eb285220	GCTGAGCGTGAGAGAGGTAT	Forward
ELFA2 - elongation factor alpha2	Zm00001eb285220	TGATGAAGTCACGGTGTCCA	Reverse
MHA2 - plasma-membrane H+-ATPase isoform MHA2	Zm00001d002006	GCTCATTGAGAAGGCCGATG	Forward
MHA2 - plasma-membrane H+-ATPase isoform MHA2	Zm00001d002006	CAGCAATACCGATGTCAGCC	Reverse
14-3-3 protein isoform GF14-6	Zm00001d003401	TCGACAAGGACGACCGAC	Forward
14-3-3 protein isoform GF14-6	Zm00001d003401	CATCTCCTCGTACCTCTCCG	Reverse
14-3-3 protein isoform GF14-12	Zm00001d025617	TGCAATGTGATCTGGACCCT	Forward
14-3-3 protein isoform GF14-12	Zm00001d025617	CTGAAACGAGGTGGGGGAT	Reverse
RBOH1 - respiratory burst oxidase1	Zm00001eb148780	ACCTGACCAGCGTGTATGAA	Forward
RBOH1 - respiratory burst oxidase1	Zm00001eb148780	TGTGTTCGAATCTTGCTGCC	Reverse
SOD1 - superoxide dismutase1	Zm00001eb008850	TTCACTTCTTCGAGGACCCC	Forward
SOD1 - superoxide dismutase1	Zm00001eb008850	CATTGGTAGTGTCGCCGAAG	Reverse
CAT1 - catalase1	Zm00001d014848	ACCCATGAACACCGTACCAT	Forward
CAT1 - catalase1	Zm00001d014848	TCGATCTTACATGCTCGGCT	Reverse
APX1 - ascorbate peroxidase homolog	Zm00001eb397180	GCCACAATGCATCCTACGAG	Forward
APX1 - ascorbate peroxidase homolog	Zm00001eb397180	GACGACCACAATAGCTGCTG	Reverse
GSR1 - glutathione reductase1	Zm00001eb004790	TTGGAATACACCGGCAGAGT	Forward
GSR1 - glutathione reductase1	Zm00001eb004790	GAAAATAGCTCGGCGGACAG	Reverse
CKO3 - cytokinin oxidase 3	Zm00001eb363220	GAGCTCCATCCGCAGATTTG	Forward
CKO3 - cytokinin oxidase 3	Zm00001eb363220	AGACGGTGCTGAAGAAAGGA	Reverse
CRR1 - cytokinin response regulator1	Zm00001eb066570	CGTCGTCATCATGTCATCGG	Forward
CRR1 - cytokinin response regulator1	Zm00001eb066570	CCTAACATAGCACCACACGC	Reverse
PIN1 - PIN-formed protein1	Zm00001eb372180	CAGTCATCTTCGGCATGCTC	Forward
PIN1 - PIN-formed protein1	Zm00001eb372180	CCCCTAGTCTGTCTCCTCCT	Reverse

Gene	Locus	Sequence 5’ → 3’	
PIN3 - PIN-formed protein3	Zm00001eb194640	GTTGTACGTGGCGATGATCC	Forward
PIN3 - PIN-formed protein3	Zm00001eb194640	CAGGTTCATGGTGTAGGGGT	Reverse
ABP1 - auxin binding protein1	Zm00001eb137840	GTCTGGGATGAGGACTGCTT	Forward
ABP1 - auxin binding protein1	Zm00001eb137840	TTTCGCGAGCCACAATACAG	Reverse
GA20OX3 - gibberellin 20-oxidase3	Zm00001eb145600	GGTGATCAGGTGGCAGACAT	Forward
GA20OX3 - gibberellin 20-oxidase3	Zm00001eb145600	AAGAAAAGAACACCAGGCCG	Reverse
GA2OX1 - gibberellin 2-oxidase1	Zm00001eb282550	AACCACTACCCTCCACCATG	Forward
GA2OX1 - gibberellin 2-oxidase1	Zm00001eb282550	ATCGTTTGATCGGAGCAAGC	Reverse
EREB10 - AP2-EREBP-transcription factor 10	Zm00001eb250540	TGTTGCACACCTAGCTAGCT	Forward
EREB10 - AP2-EREBP-transcription factor 10	Zm00001eb250540	TGTACTCCTGTTGTCGCCAT	Reverse
BRL1 - bri1-like receptor kinase1	Zm00001eb309030	AACAACCTCATCTGGCTGGA	Forward
BRL1 - bri1-like receptor kinase1	Zm00001eb309030	TGCTTCCCTGAAACAATGCC	Reverse
BSK1 - brassinosteroid-signaling kinase1	Zm00001eb402560	ACACCCTCTTTCTCCCATGG	Forward
BSK1 - brassinosteroid-signaling kinase1	Zm00001eb402560	AAGCAAAGTCCCCACGTTTC	Reverse
CDPK1 - calcium dependent protein kinase1	Zm00001eb227950	CTTGTTTGTGATGCCCCTCC	Forward
CDPK1 - calcium dependent protein kinase1	Zm00001eb227950	TCAACAAAAGACTGCCCACG	Reverse
CAL1 - calmodulin1	Zm00001eb291330	CATCCTCGACATGTTCTGCG	Forward
CAL1 - calmodulin1	Zm00001eb291330	GTAGAGGTACGGCAGGTTGA	Reverse
CAP1 - calcium pump1	Zm00001eb355420	GCGCTAACATTTGACTCCGT	Forward
CAP1 - calcium pump1	Zm00001eb355420	TTGTTTTGGCGTTAGGGTGG	Reverse
CAX1 - calcium exchanger1	Zm00001eb345450	ACTGTGGGTGGACTGTTGAA	Forward
CAX1 - calcium exchanger1	Zm00001eb345450	GCCACCAAAGAAAAGGGAGG	Reverse
KCH1 - potassium channel 1	Zm00001eb158420	GGTCGCCAAGAGATCTAGCT	Forward
KCH1 - potassium channel 1	Zm00001eb158420	TCGAGGGCTCACTGTTGAAT	Reverse
PIP1b - plasma membrane intrinsic protein1	Zm00001eb249940	CTACACCGTCTTCTCAGCCA	Forward
PIP1b - plasma membrane intrinsic protein1	Zm00001eb249940	AGTAATGGGGATGGTGGCAA	Reverse
SLAC1 - slow anion channel-associated1	Zm00001eb073420	ATGGGATTCGGCAGACAGAA	Forward
SLAC1 - slow anion channel-associated1	Zm00001eb073420	GAAATATGGCGCCACTGGAG	Reverse
SUS1 - sucrose synthase1	Zm00001eb392880	ACCACATCGACCCTTACCAG	Forward
SUS1 - sucrose synthase1	Zm00001eb392880	ACTTCTCCTCGATACGCTGG	Reverse
IDH1 - isocitrate dehydrogenase1	Zm00001eb359420	CGACTTCGCTCTCAAACTCG	Forward
IDH1 - isocitrate dehydrogenase1	Zm00001eb359420	TCGGTGTTCAGGTAATGGCT	Reverse
MDH1 - malate dehydrogenase1	Zm00001eb344340	TCTGCCACTCTTCTCACAGG	Forward
MDH1 - malate dehydrogenase1	Zm00001eb344340	CAACCTCTGTCCCACCATCT	Reverse
PEP1 - phosphoenolpyruvate carboxylase1	Zm00001eb383680	AGATGTACAACGAGTGGCCA	Forward

Gene	Locus	Sequence 5' → 3'	
PEP1 - phosphoenolpyruvate carboxylase1	Zm00001eb383680	CCACAAGCAGCTCGTCATAC	Reverse
GOGAT1 - glutamate synthase1	Zm00001eb360480	GGAAAACTGGGCGGAACTTT	Forward
GOGAT1 - glutamate synthase1	Zm00001eb360480	CTCTCAGTGTTGCGACGATG	Reverse
ZmISCA1 – iron sulfur cluster assembly 1	Zm00001eb126830	TTCTTGTGTCTGTTGCGTCG	Forward
ZmISCA1 – iron sulfur cluster assembly 1	Zm00001eb126830	CGAGGTCTTAAATCGCGAGC	Reverse
ZmISCA2 – iron sulfur cluster assembly 2	Zm00001d049325	ACCAGATACCGAAGCCGTAG	Forward
ZmISCA2 – iron sulfur cluster assembly 2	Zm00001d049325	GTAAACGGTGGCGTGATGAG	Reverse
CRY1 - cryptochrome1	Zm00001eb244770	TCACTGCAGATTTTGGCCAC	Forward
CRY1 - cryptochrome1	Zm00001eb244770	CCGTCAACTGTCTCTTCCCT	Reverse
CRY2 - cryptochrome2	Zm00001eb081200	GTCCTGTTCCCTCAAGAGCT	Forward
CRY2 - cryptochrome2	Zm00001eb081200	GACCTCTGTTCCAGCTCTGT	Reverse
PHYA1 - phytochromeA1	Zm00001eb055460	AAGAGCAGATGAGGCAGGTT	Forward
PHYA1 - phytochromeA1	Zm00001eb055460	CAGCACAAACTCAGCCATGT	Reverse
PHYB1 - phytochromeB1	Zm00001eb014850	ACCCCACCTAACTTTCTCGG	Forward
PHYB1 - phytochromeB1	Zm00001eb014850	GTGCACTGAAAACAGCCTCA	Reverse
